# Pre- and Postnatal Damage to the Retro-Geniculate Visual Pathways Cause Retinal Degeneration Predictive for Visual Function

**DOI:** 10.3389/fnhum.2021.734193

**Published:** 2021-10-26

**Authors:** Finn Lennartsson, HannaMaria Öhnell, Lena Jacobson, Maria Nilsson

**Affiliations:** ^1^Diagnostic Radiology, Department of Clinical Sciences, Lund University, Lund, Sweden; ^2^Center for Medical Imaging and Physiology, Skåne University Hospital, Lund, Sweden; ^3^Ophthalmology, Department of Clinical Sciences, Skåne University Hospital, Lund University, Lund, Sweden; ^4^Section for Eye and Vision, Department of Clinical Neuroscience, Karolinska Institutet, Stockholm, Sweden; ^5^Unit of Optometry, Department of Clinical Neuroscience, Karolinska Institutet, Stockholm, Sweden

**Keywords:** cerebral visual impairment (CVI), optic radiation, retinal degeneration, plasticity, visual system, brain development, optical coherence tomography, visual field defect

## Abstract

To increase the understanding of the relationship between structure and function in individuals with damage to the brain from different stages of maturation of the visual system, we examined 16 teenagers and young adults. We used diffusion-weighted magnetic resonance imaging (MRI) and fiber tractography of the optic radiation (OR) and optical coherence tomography (OCT) of the peripapillary retinal nerve fiber layer (pRNFL) and the ganglion cell layer + inner plexiform layer (GC+IPL) in the macula. Visual field (VF) function was assessed with the Humphrey Field Analyzer (HFA). Injuries to the immature OR were associated with thinning of the pRNFL and GC+IPL, and corresponding VF defects irrespectively of timing of the lesion. However, in cases with bilateral white-matter damage of immaturity (WMDI) we noticed a well preserved central VF despite a very thin GC+IPL. We speculate that this is due to plasticity in the immature visual system. Similar results were not noticed among cases with unilateral damage, acquired pre- or postnatally, in which the central VF was affected in most cases. OCT has proved to be a valuable targeted tool in children with damage to the retro-geniculate visual pathways, and that focal thinning of the GC+IPL predicts VF defects. This brief research report includes a review of four previously published papers. In addition, we present one new case and apply a recently developed classification system for CVI. The classification was applied on cases with bilateral WMDI to investigate its relation to retinal structure.

## Introduction

The increased survival rate of infants with critical illness prenatally or in the neonatal period, prematurely born or born at term, and of children with later acquired brain injury, has led to an increase in the prevalence of cerebral visual impairment (CVI) in children (Hatton et al., 2007; Ozturk et al., 2016). Early identification of children with CVI is the prerequisite for early intervention (Kooiker et al., 2020). The development of protocols to assess visual function early in life, of methods to document visual attention based on eye tracking tasks (Kooiker et al., 2016), and of structured history inventories are essential in the identification of children with CVI (Houliston et al., 1999; Hellgren et al., 2020). Sakki et al. (2021) recently presented a classification system, grading perception, visuomotor, and visual acuity dysfunction, in CVI. A similar severity gradient was shown in co-occurring cognitive and neurodevelopmental deficits. The primary cause of CVI in children is damage affecting the retro-geniculate visual system (pathways and processing structures). Large cupping of normal-sized optic disks associated to white-matter damage of immaturity (WMDI) was described in the 1990’s. Retrograde *trans-*synaptic degeneration (RTSD) of ganglion cell axons was suggested as the cause of this ocular sign of interruption of the optic radiation (OR) (Jacobson et al., 1997). Not only WMDI, a pattern of lesion arising in the early third trimester, but also brain malformations from the first or second trimester, and focal infarcts and hypoxic-ischemic encephalopathy from term age and later acquired lesions of multiple etiologies, may cause CVI.

New imaging techniques to map the primary injury and its relation to the retro-geniculate visual pathways and the possibility to document thinning of the macular ganglion cell layer caused by RTSD are tools that may facilitate identification of children with injuries to the retro-geniculate visual system. Structural evidence of injury to the visual pathways and secondary retinal degeneration may predict visual field (VF) defects and pose a high risk of visual dysfunction within the spectrum of CVI. However, secondary loss of ganglion cells may not be present in individuals diagnosed with CVI because of visual cognitive-perceptual dysfunction only, presenting with normal visual acuity and normal VF function.

### Evaluation of the Visual Structure and Function With Magnetic Resonance Imaging and Optical Coherence Tomography

Contrary to cerebral palsy (CP), correlations between findings on magnetic resonance imaging (MRI) and visual dysfunction has not been investigated extensively in CVI caused by early brain injuries. Injuries to the retro-geniculate visual pathways can be evaluated on MRI and include lesions in the OR or the visual cortex. Injuries to the immature OR are commonly caused by WMDI (Flodmark et al., 1989), focal infarcts (Mercuri et al., 1996) or watershed infarcts. Brain malformations can also involve the OR (Jacobson et al., 2010). However, the full extent of lesion involvement is difficult to judge on conventional MRI but can be improved with diffusion-MRI fiber tractography (Lennartsson et al., 2014, 2018; Merabet et al., 2016). With streamline fiber tractography, the OR can be generated by seeding streamlines in the lateral geniculate nucleus (LGN) and using ipsilateral targets in the medial occipital lobe (Lennartsson et al., 2014, 2018). The OR-tract can be evaluated by comparison to the expected anatomy of the geniculo-striate projections. Quantitative parameters of the OR-tract can be analyzed, e.g., to infer on the mechanisms of injuries. Associated lesions to primary injuries in the immature retro-geniculate visual pathways include injuries in the basal ganglia and thalamus, most notably the LGN (Uggetti et al., 1996), and are primary or secondary, depending on the etiology of the early brain lesion. However, involvement of these structures correlates strongly with visual impairment (Mercuri et al., 1997). Thinning of the optic nerves, the optic chiasm and optic tracts indicate secondary neurodegenerative injuries.

Optical coherence tomography (OCT) is a relatively new technique but has been heavily implemented in ophthalmology due to the valuable information it provides. Scanning of the retinal structures only takes seconds and is non-invasive. Minor participation from the patient is required. The patient needs to sit in a head-and-chin rest and keep fixation stable during the measurement. The measurement gives 3-dimensional information of the retinal structures and layer thicknesses and volume parameters with a resolution of 3–5 μm. Several studies have shown that brain injuries affecting the visual pathways cause ganglion cell + inner plexiform layer (GC+IPL) and peripapillary retinal nerve fiber layer (pRNFL) thinning and that the pattern of the GC+IPL correlates well with the location and extent of the brain injury and predicts the pattern of the VF defects (Jindahra et al., 2009; Keller et al., 2014). Despite this, OCT is not well explored in pediatric neuro-ophthalmology. Instead, the clinicians often need to rely on other ophthalmic examinations and MRI. One important tool is VF examination, but to achieve a reliable result when examining children or individuals with cognitive and physical difficulties can be challenging. The examination is time-consuming and demanding. Which method to choose needs to be adapted in relation to age and ability to co-operate. The level of participation can influence the result and lead to a high test-retest variability. An objective measure with high repeatability, like OCT, that strongly correlates with VF function is therefore valuable.

### Aim

To increase the understanding of the relationship between structure and function in individuals who present with visual impairment, caused by damage to the brain from different stages of maturation of the visual system, we examined teenagers and young adults with pre- or perinatal brain damage. We also examined a few individuals with brain damage acquired later in childhood. This text is a review of four published studies (Lennartsson et al., 2014, 2018; Jacobson et al., 2019, 2020) in which we analyzed VF function in relation to GC+IPL topography. In addition, we looked at the relation between the severity of CVI and the secondary retinal degeneration presented under “Additional analysis and one additional case.” We aim at highlighting OCT as a tool to identify children at risk for VF defects, and for CVI, early in life to make adequate habilitation possible.

## Review of Methods

### Study I–IV

The basis for our investigations has been the structuro-functional organization of the visual system, specifically the retinotopic organization of retino-striate visual pathways (Wärntges and Michelson, 2014). This has made it possible to link primary retrogeniculate injuries with secondary retinal structural changes, and their resulting VF deficits.

**FIGURE 1:**
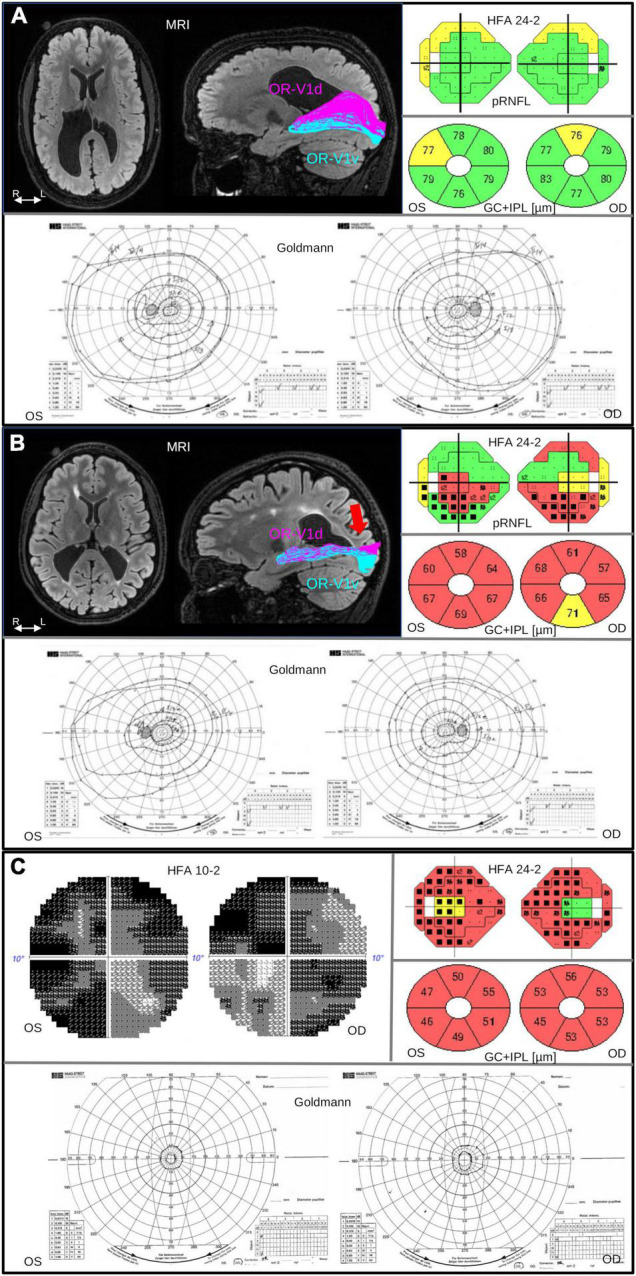
Brain injuries were examined with MRI, with salient findings illustrated on axial FLAIR image (left). Injuries to the OR were investigated with fiber tractography, with results overlaid on a sagittal FLAIR image (right), dividing the OR into parts OR-V1d (magenta) and OR-V1v (cyan) projecting to the dorsal (V1d) and ventral V1 (V1v) fMRI activation maps, respectively, described in Lennartsson et al. (2018). The pRNFL and GC+IPL thickness was measured with OCT. The pRNFL result is visualized in colors together with the results from VF examination using HFA 24-2 and GC+IPL is shown in donuts below. Color code green indicate normal, yellow; borderline and red; abnormally thin. The color codes are automatically generated and based on the reference data in the software of OCT. Visual field function was examined with Humphrey Field Analyzer (HFA) and Goldmann perimetry. The colored visual field maps show VF sensitivity (Sita Fast 24-2) in relation to corresponding pRNFL thickness. Normal VF sensitivity is represented by small single black dots, defects are shown as gray or black squares (black = deeper defect). Goldmann was performed in order to examine the peripheral VF. **(A)** Case 2 in Table 1: 19-year-old, born at GA 26 weeks, with bilateral perinatally acquired WMDI (R > L). Suffering from mild left-sided spastic CP, and dorsal stream dysfunction. VA is 1.0 (logMAR 0.00), no strabismus, and sub-grouped as CVI A1. MRI image shows bilateral WMDI (R > L) with periventricular WM reduction, especially posteriorly and on the right side. Fiber tractography shows normal ORs bilaterally, here illustrated with the right OR with separation of the OR-V1d and OR-V1v, dominating the superior and inferior portions of OR, respectively. OCT showed pRNFL and GC+IPL thickness values within normal range. The VF sensitivity and the outer borders were normal. **(B)** Case 7 in Table 1: 25-year-old woman, born at GA 33 + 0. She presented with early onset left esotropia and nystagmus. At 4 years of age VA was subnormal in both eyes and large cupping of the optic disks was noted. She has no CP. VA 0.63 (logMAR 0.20), and dorsal and ventral stream dysfunction and CVI A2. MRI image shows bilateral WMDI with WM reduction of, especially posteriorly, and extensive gliosis in the periventricular WM. Results of the left OR (right) shows abnormal appearance of the OR-V1d projections, sharing the space normally solely occupied by the OR-V1v projections, and fanning in the subcortical white matter (red arrow), presumably the location of the transient developmental subplate. OCT showed reduced pRNFL thickness, most pronounced in the superior quadrants, and globally thin GC+IPL with asymmetric thickness between the superior and inferior hemifield. HFA 24-2 shows reduced sensitivity in the inferior hemifield. Goldmann demonstrate bilateral homonymous inferior quadrant dysopias sparing the VF within 10°. **(C)** Case 5 in Table 1: 30-years old woman born at GA 29 weeks, suffered from ultrasound-verified bilateral intraventricular hemorrhages (grade 3) day 3 post-partum. She appeared to be severely visually impaired already as an infant and had early-onset exotropia and nystagmus and difficulties to maintain fixation. VA 0.25 (logMAR 0.70), and severe dorsal and ventral stream dysfunction. She has left-sided mild spastic CP, a developmental disorder and CVI B. OCT demonstrates severe thinning of both pRNFL, except in the temporal portion and extremely thin GC+IPL with only minimal asymmetry. Goldmann and HFA demonstrate severe concentric VF restriction (tunnel vision) with total loss of function in the periphery. HFA 24-2 shows some sparing of the infero-nasal part of the VF. HFA 10-2 shows minimal function also in the most central part of the VF. OR, optic radiation; R/L, right/left side; WM, white matter; WMDI, WM damage of immaturity; V1, primary visual cortex; R/L, right/left side; VF, visual field; VA, visual acuity; pRNFL, peripapillary retinal nerve fiber layer; HFA, Humphrey Field Analyzer; FLAIR, fluid-attenuated inversion recovery; GC+IPL, ganglion cell + plexiform layer.

Diffusion-weighted MRI (dMRI) and fiber tractography was used to investigate the OR and to assess lesion involvement to the tract (studies I–IV) (Lennartsson et al., 2014, 2018; Jacobson et al., 2019, 2020). In study II, our investigations were refined by inclusion of retinotopic functional MRI (fMRI) mapping. This made it possible to map the location of the cortical visual areas and separate OR into its ventral and dorsal parts of connections to the upper and lower VFs, respectively (Figure 1).

**TABLE 1:** Cases in the current studies are indexed 1–16.

**CASE ID**	**Previously**	**Gestational**	**Lesion**	**No/unilateral/**	**BCVA RE/LE**	**Mean GC+IPL**	**GC+IPL**	**VFI%**	**CVI**
	**published**	**age at lesion**	**pattern on**	**bilateral CP**		**μmRE/LE**	**asymmetry m**	**RE/LE**	**grading**
		**(weeks)**	**MRI (side/s)**	**(GMFCS I-V)**			**μm RE/LE**		
1	S3 (Figure 1 Case A)S4 (Figure 1 Case 1)	=20 GW^*a*^	MD (R)	U CP (I)	1.25/1.25	83/82	28/31	84^CD^/90	NA
2	S1 (Figure 7)S2 (Figure 2 Case C)S4 (Figure 2 Case 9)	26-28 GW^*b*^	WMDI (B, R>L)	U CP (I)	1.0/1.25	79/78	5/4	99/99	A1
3	S1 (Figure 4)S2 (Table 2 Case D)S4 (Figure 2 Case 3)	26-28 GW^*b*^	WMDI (B)	U CP (II)	1.0/0.63	71/67	14/12	92/91	A2
4	S1 (Figure 6)S2 (Figure 1)S4 (Figure 2 Case 10)	26-28 GW^*b*^	WMDI (L)	B CP (I)	0.63/1.0	80/84	5/5	100/100	A1
5	No	28-32 GW^*b*^	WMDI (B)^*^	U CP (I)	0.2/0.25	52/50	11/9	38^CD^/45^CD^	B
6	S4 (Figure 2 Case 7)	28-32 GW^*b*^	WMDI (L)	U CP (I)	1.0/1.0	67/70	3/5	98/98	No CVI
7	S1 (Figure 3)S2 (Figure 2 Case B)	32-34 GW^*b*^	WMDI (B)	no CP	0.65/0.32	65/65	10/11	81/88	A2
8	S1 (Figure 2)S2 (Figure 2 Case A)S4 (Figure 2 Case 4)	32-34 GW^*b*^	WMDI (B)	B CP (III)	0.4/0.65	68/72	13/11	88/81	A2
9	S1 (Figure 1)S2 (Table 2 Case A)S4 (Figure 2 Case 5)	32-34 GW^*b*^	WMDI (B)	B CP (II)	1.0/1.0	58/58	15/12	77/74	A2
10	S1 (Figure 5)S2 (Table 2 Case E)	26-28 GW^*a*^	WMDI (B)	no CP	1.0/0.8	67/64	6/3	98/97	A2
11	S3 (Figure 1 Case B)S4 (Figure 2 Case 2)	26-28 GW^*a*^	WMDI (R)	U CP (I)	0.63/0.63	67/69	35/31	54^CD^/55^CD^	NA
12	S4 (Figure 2 Case 8)	>34 GW^*b*^	MCA infarct (L)	U CP (I)	1.0/0.5	86/87	5/5	97/98	NA
13	S3 (Figure 1 Case C)	>34 GW^*a*^	Watershed infarcts (B, L>R)	no CP	0.9/0.8	76/77	9/8	69^CD^/64^CD^	NA
14	S3 (Figure 1 Case D)S4 (Figure 2 Case 6)	1.5 years	PCA infarcts (B, R>L)	U CP (I)	1.25/1.0	73/68	22/32	23^CD^/27^ CD^	NA
15	S3 (Figure 1 Case E)	4 years	Hemorrahgic AVM (L)	no CP	1.25/1.6	88/89	11/10	83/77	NA
16	S3 (Figure 1 Case F)	13 years	Traumatic (L)	no CP	1.25/1.25	88/89	10/11	86^CD^/86^CD^	NA

*Previous studies are marked, Study 1 = S1 (Lennartsson et al., 2014), Study 2 = S2 (Lennartsson et al., 2018), Study 3 = S3 (Jacobson et al., 2019), Study 4 = S4 (Jacobson et al., 2020). The injury pattern on MRI is described as MD, WMDI, infarcts of a vascular territory, watershed infarcts, hemorrhagic AVM, or trauma. The lesion side is noted as right/left or bilateral. If a bilateral lesion has a clear side-dominance, then this is stated.*

*No MRI was available for Case 5, but neonatal ultrasound showed bilateral intraventricular hemorrhage, a salient finding of WMDI. The lesion pattern can estimate the gestational age at lesion which then indicates, in each individual, if the injury is ^*a*^prenatal or ^*b*^perinatal. In cases with CP, this is noted with unilateral/bilateral and the GMFCS I-V. Visual acuity, OCT measurements of the GC+IPL, and VFI are reported. The GC+IPL asymmetry refers to the difference between the thickest and thinnest GC+IPL sector. For comparison, the mean GC+IPL thickness ranged between 77–94 μm and the asymmetry ranged between 2–7 μm in the control group.

*^CD^Focal VF defects within the 4 central test locations, as examined with HFA 24-2, are marked in the column reporting VFI. CVI grading is done according to Sakki et al. (2021) when applicable.*

*GW, gestational week; MD, malformations of cortical development; WMDI, white-matter damage of immaturity; M/PCA, middle/posterior cerebral artery; AVM, arterio-venous malformations; R/L, right/left; B, bilateral; U, unilateral; CP, cerebral palsy; GMFCS, Gross Motor Function Classification System; NA, not applicable; BCVA, best corrected visual acuity (decimals); RE/LE, right/left eye; GC+IPL, ganglion cell + inner plexiform layer; VFI, visual field index; OCT, optical coherence tomography; CVI, cerebral visual impairment; HFA, Humphrey Field Analyzer.*

Optical coherence tomography (OCT) was used to measure the pRNFL (study I) and GC+IPL in the macula (studies II–IV). Visual field function was assessed with the Humphrey Field Analyzer (HFA) (studies I–IV) using Sita Fast 24-2 (testing 24° temporally and 30° nasally) and Goldmann perimetry (study I). The result from the functional and structural examinations has been mapped for each subject in order to study how the pattern of VF defects associates with the GC+IPL topography.

## Review of Results

### Study I

In our first study (Lennartsson et al., 2014), we investigated seven young adults with CVI caused by WMDI and various degrees of VF deficits with spared function in the central 5° of the VF. WMDI, particularly periventricular leukomalacia, has a predilection for peritrigoneal white matter resulting in characteristic bilateral homogenous dysopias in the lower VF, indicating involvement of the superior portion of the OR. White matter fiber tractography was used to map and assess injury involvement of the OR. OCT was used to measure the pRNFL to detect signs of RTSD in the retina after brain injuries. The study confirmed that only cases with lesional involvement of the superior portion of the OR, as assessed from white matter fiber tractography, displayed commensurate reductions in the pRNFL on OCT, and corresponding VF defects. The strong correlation between pRNFL and VF sensitivity, and the relatively thinner axon layer in the superior quadrant of the optic nerve head corresponding to the damage in the superior portion of the OR, gave evidence of the occurrence of RTSD after brain injury.

### Study II

In the second study (Lennartsson et al., 2018), we showed that, indeed, there were strict topographical and quantitative correlations between the injury in the OR and the thinning of the GC+IPL. We also found that the GC+IPL topography had a stronger predictive ability of the VF function compared to pRNFL results. Further, the ratio of the mean thickness of the GC+IPL in the superior and inferior sectors significantly correlated with the dMRI parameters of axial and mean diffusivities in the dorsal OR, but not in the ventral OR. We interpreted those results as evidence for RTSD. Moreover, with retinotopic fMRI mapping we investigated the central 11° VF. This generated coherent and reliable V1 cortical activation maps in all subjects, with no difference between subjects and controls. This confirms that all WMDI cases, including cases with injury to the OR and RTSD with thinning of GC+IPL, had apt cortical responses within the central 11° VF.

### Study III

Having identified the mechanism of RTSD in CVI caused by WMDI, we proceeded to investigate injuries with different timings. In a smaller case-study, study III (Jacobson et al., 2019), we included six young adults with pre-natal and post-natal injuries occurring at different times. Again, fiber tractography was used to identify the parts of the OR which were affected by lesions, and OCT of the GC+IPL was used to detect secondary retinal changes. In all subjects, RTSD were found to cause thinning of the neuro-retinal structure regardless of the injury timing. This study further confirmed the importance of looking for GC+IPL asymmetry to be able to predict the pattern of the VF defect based on OCT, especially in cases with a physiologic thick GC+IPL or pRNFL layer to start with, and in whom focal thinning may be missed when compared with the normative data base.

### Study IV

The convincing results, i.e., that OCT can detect focal retinal RTSD, made us consider its use as a targeting tool to identify individuals with CVI causing VF defects. We addressed this in our study IV (Jacobson et al., 2020), in which we investigated ten subjects with spastic CP, a patient group with high risk of injury to the OR. Using our established method of fiber tractography of the OR and OCT of the GC+IPL, we found, again, focal thinning of the GC+IPL in cases with damage to the OR, resulting in VF defects. In individuals without injuries affecting the OR, we found no focal GC+IPL thinning or VF defects.

Based on these results from studies I–IV, we suggested that children presenting with symptoms of brain damage should generously be examined with OCT early in life. As reliable perimetry is extremely difficult to achieve in pre-school aged children, OCT could serve as a targeted test to identify VF defects in young children (3–4 years of age) making early identification of children with CVI possible.

## Additional Analysis and One Additional Case

The material included in this additional analysis is presented in Table 1 and all subjects are linked to previous studies.

We applied the recently developed scale for subgrouping of individuals with CVI suggested by Sakki et al. (2021). The subgrouping is based on the severity of visual dysfunction: A1 with selective visual perception and visuomotor deficits, A2 with more severe visual perception and visuomotor deficits and variable visual acuity, B with lower function and significant visual acuity reduction compared with A. All individuals with bilateral WMDI from studies I and IV (eight) were subgrouped.

To increase the understanding of the relationship between structure and function we have added one new individual (Case 5 in Table 1) with bilateral WMDI and more severe visual impairment than the 15 individuals included in the four published studies. The findings are demonstrated in Figure 1C, and are further elucidated in the discussion. Results are summarized in Table 1 and the participation of each case, in more than one study, is stated.

### Results

**FIGURE 2:**
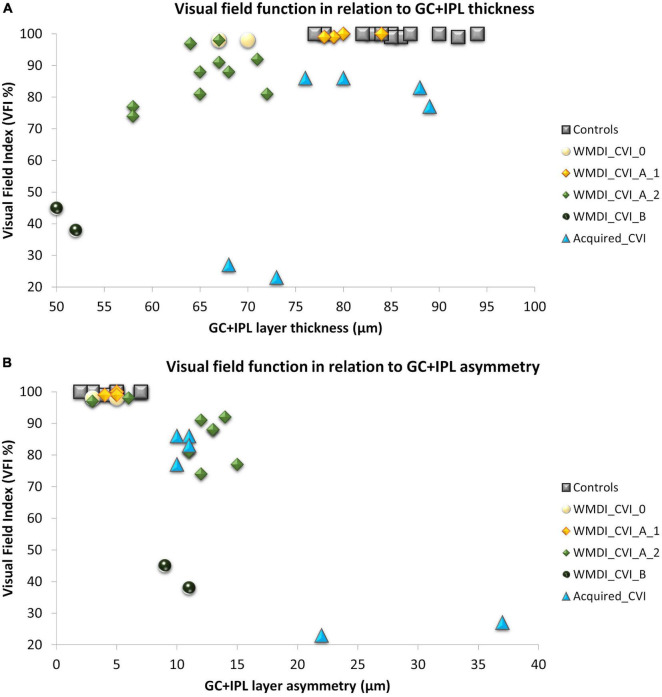
**(A)** A strong association was seen between retinal structure and function in patients with WMDI. In relation to the range of controls, one WMDI without CVI showed a thin GC+IPL, all grouped as A1 had normal GC+IPL. All subjects in A2 had a thin GC+IPL. Among the three subjects with acquired CVI, one subjects had a thin GC+IPL. **(B)** All subjects with GC+IPL asymmetry had reduced VF sensitivity. CVI subgroups A1 was separated from A2 and B by GC+IPL asymmetry in all but one case graded with A2 who did not have any VF defect or GC+IPL asymmetry. WMDI, white matter damage of immaturity; CVI, cerebral visual impairment; CVI A1, A2, and B are subgroups of CVI severity according to Sakki et al. (2021) “Acquired” includes subjects with CVI due to brain injury after 18 months of age. GC+IPL, ganglion cell + inner plexiform layer.

According to the CVI-classification by Sakki et al. (2021), one subject had normal visual function (A0), two were classified as A1, five as A2 and one as B. Three individuals, representative of the groups A1, A2, and B, are presented in Figure 1 with MRI (when available), HFA and OCT measurements. Three individuals with unilateral (two) or bilateral (one) homonymous VF defects caused by injuries acquired later in life, Study III, were compared to these nine individuals with WMDI (Figure 2). These three subjects were not graded, as such children were excluded from the study by Sakki et al. (2021).

Thereafter, correlations between retinal structure and function were plotted with respect to the CVI subgrouping, see Figure 2. For comparison, a reference group of 12 healthy young adults was included in the plot. They had all been part of previous studies from our research group (Lennartsson et al., 2014; Nilsson et al., 2016). Inclusion criteria were birth at term, no history of ocular disease and absence of visual complaints. As shown in our previous studies, the GC+IPL thickness and GC+IPL asymmetry (defined as the difference between the thickest and the thinnest sector) in relation to the VF index (VFI) is a valuable way of describing retinal structure and function in relation to the location and extent of damage to the OR. Therefore, these parameters were applied for this purpose.

As shown in our previous studies, there is a strong correlation between average GC+IPL thickness and VFI in the WMDI group (Figure 2A). Two subjects in the WMDI group had a GC+IPL thickness within the range of controls and seven had a thinner. Out of these seven, two subjects had a thin GC+IPL layer, but no asymmetry and no focal VF defects.

In the three individuals with injuries acquired later in life, the average GC+IPL was close to, or within the range of controls, in two subjects. However, all three showed a GC+IPL asymmetry outside the range of controls and they all had focal VF defects (Figures 2A,B).

In WMDIs with no or CVI grade A1, focal VF defects were absent and no GC+IPL asymmetry was noticed although, one subject had generally thin GC+IPL (Figures 2A,B). Among those with CVI grade A2 and B, all subjects had reduced GC+IPL thickness. All but one had GC+IPL asymmetry, this individual has a VF defect only in the periphery, in opposite to the others. The CVI classification separated the subgroups and A2 and B showed to be related to increased GC+IPL asymmetry and focal VF loss (Figure 2B).

## Discussion

We have, in a series of studies, shown that primary injuries in the OR cause secondary degeneration in the retina, and associated VF defects. The topographically correlating injuries in the retino-striate system are evidence of RTSD and occur irrespectively of the timing of the injuries. Conversely, OCT of the GC+IPL can predict injuries to the OR and should be considered as an early targeted investigation in individuals with CVI, or risk of CVI.

Although retinal RTSD was seen in all individuals with damage to the OR, regardless of timing, there were some differences between groups (Figures 2A,B). All bilateral WMDIs had their best-preserved vision in the central VF (Lennartsson et al., 2014). They all had a generally thin GC+IPL layer, and those with moderate VF damage had GC+IPL asymmetry. Among cases with acquired injuries, the average GC+IPL thickness was within the range of controls in two out of three subjects, instead they had a large GC+IPL asymmetry and focal, complete VF defects engaging also the most central part of the VF (Jacobson et al., 2019). Further, GC+IPL asymmetry seems useful to detect focal VF defects in this whole group of patients but also to recognize individuals with CVI grade A2-B related to WMDI.

One subject with bilateral WMDI and CVI grade B had a very thin GC+IPL and severe VF loss without any pronounced GC+IPL asymmetry, most likely due to the “floor effect”. This effect is a limitation in measurements of the GC+IPL and pRNFL thickness using OCT and can be described as the point when no further structural loss can be detected despite loss of VF function. Attempts to estimate the floor effect has mainly been done in patients with glaucoma. Bowd et al. (2017) found a floor effect for GC+IPL between 31 and 45 μm and Mwanza et al. (2015) between 49 and 65 μm for the pRNFL. Both studies were based on examination of patient with moderate to advanced glaucoma and used different OCT machines. In the context using OCT to reveal VF deficits related to brain injury it is important to consider that severe loss of axons due to bilateral extensive cerebral damage is unlikely to cause asymmetry, but rather a markedly reduced average thickness in relation to the normality database.

Key elements for the organization of the visual system are the aggregation of the GC projections into eye-specific layers in the LGN, largely achieved by midgestation (Hevner, 2000) and is believed to be a prerequisite for the formation the eye-specific ocular dominance columns in the striate cortex during the second half of gestation (Penn and Shatz, 1999), following the invasion of geniculo-striate axons in the cortical plate at around 23–25 GW (Kostović and Judaš, 2010). This is largely achieved by the preservation of the retinotopic map and has been linked to retinotopically coordinated, spontaneous waves of firing in the retinal ganglion cells over the retina before (Shatz, 1996), and sensory driven activity after, opening of the eyes (Vanhatalo and Kaila, 2006). An adult-like pattern of retino-cortical connection is essentially established at time of normal birth (Kelly et al., 2014). In the fetal human retina, the GC count in the optic nerve shows an elevated plateau from midgestation up until around 30 GW, suggesting an overproduction and elimination of the GC in the immature retina (Provis et al., 1985). In parallel, there is a migration of the inner retinal layers and creation of the foveal pit, a process that extends well into the second year of life (Dubis et al., 2012). Taken together, this indicates that dynamic processes are present in the immature visual system, throughout the perinatal and long into the post-natal period.

There is evidence of a better visual recovery after injuries to the immature visual system than after injuries sustained later in life, attributed to its superior potential for plasticity. Our experience is that bilateral WMDIs have spared central VF function within 5° based on perimetry outcome, and spared function within 11° in retinotopic mapping with fMRI (Lennartsson et al., 2018), also when the damage to the OR is comprehensive. When the central VF, in such cases is affected, a total constriction of the VF needs to be suspected. The well-preserved central VF function in bilateral WMDIs, despite thin GC+IPL, may be explained by an early re-organization of the ganglion cells. Could it be, that the normal migration of ganglion cells towards the mid-periphery becomes inhibited to cover up the central vision on behalf of the more peripheral? Interestingly, a time-dependent relationship also exists between injuries during different stages of gestation (Jacobson et al., 2010) and is linked to the different developmental stages of the immature visual system (Guzzetta et al., 2010). In our studies, we found indications of plasticity in the immature OR (Lennartsson et al., 2018), with displacement of connections to dorsal striate cortex to the space normally solely occupied by connections to the ventral striate cortex (Figure 1B). Could it be the result of a compensatory mechanism to preserve the VF map by keeping connections that are normally pruned away? Perhaps as a result of retinal plasticity with an expansion of the receptive fields, and that the plasticity processes in the OR concur to promote central VF function and coverage? This could potentially be addressed by investigating the population receptive fields with functional MRI to map the receptive fields on the visual cortex (Dumoulin and Wandell, 2008).

Altogether, this review includes the results from examination of 15 teenagers/young adults. Only individuals with the intellectual and motor prerequisites to maintain fixation during OCT and capacity to carry out standardized perimetry were invited. One of these 15 did not have CVI. Of the 14 individuals with CVI, three were subtyped as A1 and eight as A2 (Sakki et al., 2021) and three had CVI caused by later acquired retro-geniculate lesions and therefore not sub-grouped. Thus, the studied individuals are not representative of all young adults with pre- or postnatal brain damage as individuals with more severe functional deficits were not invited. We have added one case (Figure 1C, case 5 in Table 1) with bilateral WMDI and CVI subgroup B, not published before, to highlight the need for further studies to understand the retinal consequences of extensive damage to the immature visual brain. This case is an example of how injury to the immature brain, can cause total loss of the VF function, sparing only the most central part, however, with extremely reduced sensitivity and no high-resolution acuity. Such severely restricted peripheral fields, known as tunnel vision, have mainly been described associated to retinitis pigmentosa and glaucoma. However, we speculate that the few survivors among the GCs in case 5, due to plasticity, and not just by chance, are spared for the very central VF. Van Hof-van Duin and Mohn (1984) described tunnel vision, assessed with confrontation, in association with perinatal hypoxic brain damage in five children.

For individuals with signs of more severe brain damage, such as CP Gross Motor Function Classification System (GMFCS) IV and V and inability to maintain fixation during OCT, there is a need for further development of OCT devices to capture reliable measurements of the GC+IPL. In these groups the risk for severe retinal degeneration secondary to damage to the OR, and severe visual impairment may be considerable, however often underestimated.

A limitation of our studies is the low number of included individuals, and that most of them represent individuals with less extensive brain damage. Future studies with handheld OCT may increase the knowledge about the prerequisites to use vision, for example for communication in non-verbal children with severe CP.

In children with brain damage, focal or general thinning of the GC+IPL indicate deficits in VF function, and a high risk of CVI. However, normal GC+IPL does not eliminate the risk for cognitive-perceptual visual impairment, CVI subgroup A1. OCT has proved to be a valuable tool in clinical pediatric ophthalmology, not only in children with diseases affecting the eye and anterior pathways, but also in children with damage to the retro-geniculate visual pathways.

## Data Availability Statement

The raw data supporting the conclusions of this article will be made available by the authors, without undue reservation.

## Ethics Statement

The studies involving human participants were reviewed and approved by the Swedish Ethical Review Authority (Dnr 2020-06677) Regional Ethical Review Authority Stockholm (Dnr 2013/1114-31/2). Written informed consent to participate in this study was provided by the participants’ legal guardian/next of kin.

## Author Contributions

FL, HMÖ, LJ, and MN contributed to the conception and design of the study, and wrote the sections of the manuscript. FL and MN performed the statistical analysis. LJ wrote the first draft of the manuscript. All authors took part of the data collection and contributed to manuscript revision, read, and approved the submitted version.

## Conflict of Interest

The authors declare that the research was conducted in the absence of any commercial or financial relationships that could be construed as a potential conflict of interest.

## Publisher’s Note

All claims expressed in this article are solely those of the authors and do not necessarily represent those of their affiliated organizations, or those of the publisher, the editors and the reviewers. Any product that may be evaluated in this article, or claim that may be made by its manufacturer, is not guaranteed or endorsed by the publisher.
